# Decisional incentive sensitivity is linked to contingency management outcome and striatal dopamine signaling in individuals with cocaine use disorder: a preliminary study

**Published:** 2026

**Authors:** Nehal P. Vadhan, Kenneth M. Carpenter, Elysia Benedict, Catherine E. Myers, Mark A. Gluck, Diana Martinez, Edward V. Nunes

**Affiliations:** 1 Northwell, New Hyde Park, NY, USA; 2 Departments of Psychiatry, Molecular Medicine and Psychology, Hofstra University, Hempstead, NY, USA; 3 Institute of Behavioral Science, Feinstein Institutes for Medical Research, Manhasset, NY, USA; 4 Department of Psychiatry, Columbia University Irving Medical Center, NY, USA; 5 Neuroscience and Surgery Institute of Delaware, Newark, DE, USA; 6 Lankenau Medical Center, Wynnewood, PA, USA; 7 VA New Jersey Health Care System, East Orange, NJ, USA; 8 Department of Pharmacology, Physiology & Neuroscience, Rutgers University-New Jersey Medical School, Newark, NJ, USA; 9 Rutgers University – Newark, Newark, NJ, USA

**Keywords:** Cocaine, Iowa Gambling task, Decision-making, Contingencies, Reinforcement, Motivation community reinforcement, Dopamine, Striatum, PET

## Abstract

**Background:**

Nontreatment-seeking individuals with cocaine use disorder (CUD) have been found to exhibit decision-making on laboratory tasks that is risky but also sensitive to monetary incentive, relative to controls.

**Objective:**

The purpose of this study was to replicate these findings in treatment-seeking individuals and explore their relationships with voucher-based treatment outcome and striatal dopamine (DA) release.

**Methods:**

This study was approved by the Institutional Review Board, and all participants provided written informed consent to participate. Eighteen briefly-abstinent individuals seeking treatment for CUD and 19 control participants were compared on performance of a modified Iowa Gambling task (mIGT) under both hypothetical and cash earning conditions. The CUD participants subsequently received Positron Emission Tomography (PET) scans with [^11^C]raclopride with methylphenidate (60 mg) challenge and then 12 weeks of Community Reinforcement Approach plus Vouchers (CRA+V) treatment.

**Results:**

On the mIGT, the CUD participants’ advantageous card selection was selectively more sensitive to the presence of a monetary incentive, relative to controls (F4,128 = 3.10, p<0.05). Among the male CUD participants, those who exhibited greater DA release in the ventral striatum (VSt), and those who responded to the CRA+V treatment, exhibited greater mIGT incentive sensitivity than those who were nonresponders (F_4,44_ = 5.84, p<0.01), and who exhibited relatively decreased VSt DA release (F_4,48_ = 2.55, p<0.05).

**Conclusions:**

Participants seeking treatment for CUD exhibited selectively increased decisional incentive sensitivity, relative to controls. For the male CUD participants, greater incentive sensitivity was associated with greater VSt DA release and better CRA+V outcome. These findings appear partially consistent with previous findings on cognition and motivation in individuals with CUD, and suggest a heuristic model connecting striatal DA, incentive sensitivity, and CRA+V outcome.

## Introduction

Cocaine use remains prevalent in the US (>2 million current users; SAHMSA, 2022) and cocaine use disorder (CUD) (>1.4 million past-year cases; SAHMSA, 2022) has been difficult to treat. Community Reinforcement Approach plus Vouchers treatment [1] is a moderately efficacious approach that shapes cocaine abstinence with monetary rewards for initial abstinence and naturalistic rewards for sustained abstinence [2–4]. However, treatment response tends to be dichotomous and established early in treatment [5], suggesting that there is differential sensitivity to these alternative reinforcers. Investigating this sensitivity is critical to increasing treatment efficacy and broadening its effectiveness.

In terms of understanding such individual differences, psychological functions that lie at the intersection of cognition, motivation and reward have been shown to relate to the outcome of voucher-based treatment [6] for CUD [7–10]. Decision-making, defined briefly as the balancing of choices that bring larger but riskier rewards with those that bring smaller but safer ones [11], is a related candidate mechanism that has been minimally explored in this regard [12].

Individuals with CUD have been repeatedly found to be risky in their decision-making on laboratory tasks involving monetary earnings and losses [e.g., 13–16] and weak in underlying stimulus-response (S-R) learning functions [17–20], relative to non-cocaine users. However, the typical use of hypothetical money in most of these studies [21] raises questions of performance motivation and ecological validity.

We [15] found that while nontreatment-seeking individuals with CUD (n=25) made riskier decisions than controls (n=19) under hypothetical earnings/losses on a modified version of the Iowa Gambling Task (IGT; [22,23], there were no group differences when actual cash earnings/losses were employed. Similarly, in other groups of substance users, monetary incentives have been found to: 1) decrease risk-taking (Balloon Analogue Risk Task; [24,25], 2) increase response inhibition (anti-saccade task; [26] and improve (via contingency management; CM) adherence to and cognitive outcome from cognitive remediation [27]. Thus, incentives can improve cognitive performance, reduce aspects of impulsivity, and catalyze the learning of more adaptive choices. However, there may be individual variability in sensitivity to incentives that have relevance for outcome prediction.

Processing of monetary rewards has been directly and indirectly found to be related to dopamine (DA) transmission in the striatum [e.g., 28–32]. Importantly, DA deficiency in the ventral striatum (VSt) in CUD participants has been repeatedly found [33] and prospectively associated to: 1) increased cocaine self-administration in the human laboratory [34], and 2) decreased response to CRA+V therapy [34,35]; both procedures leverage financial incentive to challenge decisions to use cocaine.

Studies in treatment-seekers for methamphetamine (MA) use disorder demonstrated that CM nonresponders exhibited riskier IGT card selection relative to healthy controls and CM responders [36], and that more conservative card selection was prospectively associated with MA abstinence [37]. These findings explicitly link decision-making to voucher treatment outcome, but these studies did not employ a choice-contingent monetary incentive on the IGT, nor any neurobiological measure. This limits a more comprehensive understanding of potential mechanisms.

Thus, the objectives of the current study were to analyze data from a previous study [35] to: 1) replicate and extend several studies [i.e., 15–18] by evaluating mIGT performance (with and without monetary incentive) and S-R learning performance in abstinent individuals seeking treatment for CUD, and controls; and 2) derive measures of incentive sensitivity and determine their relationship to clinical and neurobiological variables in the CUD participants (i.e., CRA+V treatment outcome, VSt DA function). We hypothesized that: 1) individuals with CUD would exhibit greater incentive sensitivity on the mIGT and weaker S-R learning performance than controls; and 2) individuals with CUD with efficacious treatment response/greater VSt DA release would exhibit greater incentive sensitivity than those with non-efficacious treatment response/lesser DA release.

## Materials and Methods

This study was approved by the Institutional Review Board of the New York State Psychiatric Institute and was carried out following the rules of the Declaration of Helsinki of 1975 [38]. All participants provided written informed consent and were compensated for their time. The submitted manuscript adheres to the ICMJE Recommendations [39]. All data were collected at Columbia University Irving Medical Center.

### Participants

Participants were males and females between 21 and 45 years old. Participants were excluded if they met criteria for any DSM-IV Axis I disorder including other substance use disorders other than CUD (for the cocaine users), and if they reported current use of psychoactive medications, and substances other than cocaine, cannabis, alcohol, nicotine or caffeine. Illicit substance use and nonuse was verified by urine toxicology tests during screening and testing. This was a sample of convenience.

### Individuals with cocaine use disorder (CUD)

These were a subset of participants seeking treatment for CUD from a previously published study [35] that received the cognitive assessment (n=18), neuroimaging (n=17) and CRA+V (n=18). Methods for participant recruitment, screening and neuroimaging are described in detail in that article and are presented briefly here.

### Control participants

Nineteen individuals without cocaine use, who were independently recruited (i.e., a different control group than from the primary study) via local newspaper, internet and flier advertisements to match the presence of alcohol and cannabis use in the CUD participants, participated in this study. Reported lifetime use of cocaine greater than 10 times was exclusionary. All participants stated that they were not seeking or engaged in treatment for any substance use disorder. They completed their participation within one week of screening and were compensated for their time.

Demographic, clinical, and substance use characteristics for both groups are presented in Table 1. The CUD group was predominantly male and of late 30s mean age, with an early onset of regular cocaine use. Continuous variables were analyzed with two-tailed independent-samples t-tests and categorical variables were analyzed with chi-square.

Significant group differences were seen for depression and impulsivity symptoms (BDI-II; EIQ; CUD > Cont), proportion of female participants (Cont > CUD) and self-reported amount of cannabis use per occasion (Cont > CUD).

### Design and procedure

#### Monitoring of cocaine abstinence

Two weeks of verified abstinence were required of the individuals with CUD prior to neuroimaging [35]; they had the option of either staying inpatient at CUMC’s Irving Research Unit (n=11) or submit to thrice-weekly outpatient urine toxicology testing (n=7). Controls’ lack of current cocaine use was verified via urine toxicology testing during screening and testing sessions.

#### Cognitive testing session

All testing took place in a quiet room (1 day prior to imaging for CUD participants); participants were given in-person instructions per task and then monitored by remote video; outpatient participants were instructed not to use any psychoactive substance on the morning of testing except regular caffeine and nicotine. All participants passed alcohol breathalyzer, field sobriety tests, spent 20–30 min completing self-report/urine tests, and displayed no behavioral signs of intoxication as noted by the experimenter; thus participant intoxication was unlikely.

### Measures (all computerized, and described briefly here)

#### Decision-making and incentive sensitivity

##### Modified Iowa Gambling Task [23]:

The mIGT task was administered under two conditions (100 trials each) to each participant: (1) hypothetical payment (Hypothetical) and (2) cash payment (Cash). Condition order was counterbalanced with an interval of 2–3 hours, and participants were blind to the repeated administration and condition type until just prior to condition onset.

Four decks of cards (A–D) were displayed and participants were credited with $10. Participants were instructed to select cards (one at a time) from any of the decks to win as much money as possible, and that some decks were better than others. Following each card selection, participants were credited with some amount of money; occasionally after a card selection, participants simultaneously won and lost some amount of money. Participants were informed that they would keep any winnings. Two of the decks (C and D) paid an average of $0.50 per card selection but were associated with an average loss of $0.25 per selection (potential net earnings=$25.00). In contrast, the other two decks (A and B) paid an average of $1.00 per selection but were associated with an average penalty of $1.25 per card (potential net loss=$25.00). These values were proportionally identical to the traditional IGT, and deck location was randomly assigned each administration. The 3 primary performance measures were: 1) card selection (number of cards selected from advantageous decks), money earned ($), and time to complete the task (sec).

#### Stimulus-Response Learning

##### Weather Prediction Task [40]:

This task measures probabilistic category learning via binary choice. Participants were instructed to predict, via keystroke, the weather (“sun” or “rain”) based on a random array of 1–3 cards (200 trials) and were given accuracy feedback. Each card was associated with each outcome with a fixed probability (e.g., card 1 was associated with sun on 80% of the trials on which it appeared and with rain on 20% of the trials on which it appeared) and the outcomes sun and rain appeared equally often [40]. The dependent measure was percent optimal responding, i.e., choosing the outcome that was most associated with each particular stimulus configuration over the course of the task (see [41]).

##### Acquired Equivalence Task [42]:

This task measures equivalence learning and learning transfer via binary choice. On each trial, 1 of 4 distinct cartoon faces were displayed (antecedents A1, A2, B1, or B2) and 2 of 4 distinct cartoon fish (consequents X1, X2, Y1, or Y2). The participant decided which fish belonged to the person (at first by guessing) with a key response and received accuracy feedback. In Acquisition stage 1 (shaping), two antecedents were each associated with a different consequent (e.g., A1→X1, B1→Y1); in Acquisition stage 2 (equivalence training) the remaining two antecedents were each associated with a stage 1 consequent (A2→X1, B2→Y1). Acquisition stage 3 (novel consequents) consisted of two antecedents trained with novel consequents (A1→X2, B1→Y2). During the Transfer phase, participants were tested (with no feedback) on all six previously-trained face-fish pairs as well as two novel (untrained) pairs (A2→X2, B2→Y2). The dependent measure for Acquisition stages 1–3 was errors to criterion, and the dependent measures for the Transfer phase was the percent of total errors made on trials that tested novel and previously-learned pairs.

#### Naturalistic behavior

##### Risk Assessment Battery (43):

This is a 24-item questionnaire that measures drug and sexual risk-taking. The total score was employed in this study.

##### Barratt Impulsiveness Scale (44):

This 30-item questionnaire measures attentional, motor, and non-planning features of impulsiveness. The total score was employed in this study.

#### Neuroimaging

See Martinez, Carpenter [35] for full details. For all subjects, [^11^C]raclopride was administered as a bolus and the PET scans were acquired on the ECAT EXACT HR+ scanner (Siemens/CTI, Knoxville, Tenn.) in three-dimensional mode over 60 minutes. All participants underwent two scans with [^11^C]raclopride: at baseline and following administration of oral methylphenidate (60 mg). A plasma sample for analysis of methylphenidate level was obtained just before the second scan. The PET data were analyzed by means of simplified tissue reference modeling [45], with the cerebellum used as a reference region to estimate nonspecific binding. The PET outcome measure was binding potential (BP_ND_) defined as BP_ND_ = f_ND_ * B_max_/K_D_ (see Supplementary File) [46]. The percent change in [^11^C]raclopride binding following methylphenidate administration was defined as (BPNDbaseline – BPNDmethylphenidate)/BPNDbaseline [47,48], with greater values indicating greater DA release. In addition to the PET scans, each participant also underwent a magnetic resonance imaging (MRI) scan (GE Signa EXCITE 3T/94 cm scanner, GE Medical Systems, Milwaukee, Wis.) for identification of the region of interest (ventral striatum).

#### Treatment

Following the PET scans, the cocaine-dependent subjects began manualized Community Reinforcement Approach plus Vouchers (CRA+V; (1); see Supplement for details). The therapy sessions were conducted twice weekly by trained therapists, with weekly supervision provided. The participants received voucher points for each urine sample that tested negative for a cocaine metabolite (i.e., benzoylecgonine). The voucher points ($0.25) were acquired on an escalating schedule that started at 10 points for the first cocaine-free sample, and each subsequent cocaine-free sample increased the voucher value by 5 points. Participants also received a bonus of 40 points ($10.00) for every three consecutive cocaine-free urine samples (equivalent to a week of abstinence). Participants could earn a maximum of $997.50 in vouchers for submitting cocaine-free urine samples at 100% of the scheduled treatment visits (36 over the course of 12 weeks).

### Data analyses

#### General approach

Performance on the three tasks was first examined as a function of group (individuals with CUD vs. controls) and trial block/type with mixed-design repeated measures Analysis of Variance (ANOVA); one ANOVA was conducted for each dependent measure. To examine the effects of sex (female vs male) on performance [49], sex was added as an additional independent factor to the above models. Significant interactions were probed with simple effect tests. Interactions involving both group and sex were not reported or probed due to the relatively small sample of females (n=3) in the cocaine-using group. For all analyses, only p-values <0.05 were considered statistically significant and Huynh-Feldt corrections were employed to correct for violations of sphericity.

See Supplementary File for task-specific data analysis characteristics.

#### Incentive sensitivity

Incentive sensitivity indices were derived by calculating the difference score between mIGT performance under the cash condition and performance under the hypothetical condition; larger scores indicated relatively better performance under the actual cash incentive (i.e., greater adaptive incentive sensitivity).

#### CUD-only analyses

Due to the relatively small sample size, the relationships of task variables to treatment response and to dopamine release were examined separately. To account for the potential influence of sex differences on task performance, these secondary set of analyses were performed only on the male participants’ data (n=15).

### Treatment response

The distribution of total CRA+V vouchers earned for the male subsample was examined to determine treatment response classification. As in [35], the distribution was bimodal (5 responders/10 nonresponders). Comparison on total voucher earnings between responders and nonresponders via univariate ANOVAs was conducted to verify subgroup assignment. The hypothesized relationships between incentive sensitivity and treatment outcome were examined by comparing these treatment subgroups on the cognitive tasks, with mixed-design repeated measures Analyses of Variance (ANOVA). Due to potential subgroup differences in cocaine use, and corresponding influences on treatment outcome, baseline frequency of cocaine use (days in the past 30) was added as a covariate.

#### Dopamine function

The distribution of striatal dopamine release for the male participants was examined to determine subgroups based on dopamine release capacity (high versus low). The distributions were bimodal (8 high/6 low). Comparison on total voucher earnings between responders and nonresponders via univariate ANOVAs was conducted to verify subgroup assignments. The hypothesized relationships between incentive sensitivity and DA release capacity were examined by comparing these DA release capacity subgroups on the mIGT indices, with mixed-design repeated measures Analyses of Variance (ANOVA).

To address potential asymmetric effects of condition order (Hypothetical first vs. Cash first) on performance, it was included as a covariate in all analyses involving the mIGT.

## Results

Statistical results are presented inline in the text for figures, and in Supplementary Tables 2–7 for other data.

### CUD and control participants

#### mIGT performance

##### Card selection (Figure 1):

There were no main effects of monetary condition (F_1,32_ = 1.83, η_p_^2^ = 0.05, p>0.05), block (F_4,128_ = 0.63, η_p_^2^ = 0.02, p>0.05), or sex (F1,32 = 2.94, ηp2 = 0.08, p>0.05), but there was a main effect of group (F_1,32_ = 5.66, η_p_^2^ = 0.15, p<0.05), with the CUD participants selecting more cards from advantageous decks on average per block than the control participants. However, there were block × group (F_4,128_ = 2.53, η_p_^2^ = 0.07, p<0.05), monetary condition × block × group (F_4,128_ = 3.10, η_p_^2^ = 0.09, p<0.05) and monetary condition × block × sex (F_4,128_ = 3.03, η_p_^2^ = 0.09, p<0.05) interactions.

The CUD participants’ card selection was more advantageous overall than the controls’ (Panel A) for blocks 2 (p<0.01) and 5 (p<0.05), with no group differences for any of the other blocks (p>0.05). For the CUD participants, card selection was more advantageous under the cash than hypothetical condition for block 2 (p<0.05), whereas there was no such difference for any block for the controls (p>0.05). Thus, the CUD participants selected more advantageously: 1) specifically for 2 blocks, relative to the control participants, and 2) under the cash contingency relative to the hypothetical contingency (for 1 of those trial blocks).

For the females (Panel B), card selection was more advantageous under the cash than hypothetical condition for block 2 (p<0.05), whereas there was no such difference for any block for the males (p>0.05). For both group comparisons, in no case was card selection significantly more advantageous under the hypothetical than cash condition (p>0.05).

##### mIGT Money earned (Supplementary Table 2A):

There were no main effects of monetary condition, block, group or sex (p>0.05). There was a block × group interaction (p<0.05), whereby the controls earned more money on the first block (p<0.05).

##### mIGT Task completion time (Supplementary Table 2B):

There was a main effect of monetary condition on completion time, with slower performance for both groups under the Cash condition, relative to the Hypothetical condition (p<0.001). There were no main effects of block, group or sex (p>0.05), and no other interactions (p>0.05).

#### Weather Prediction task (Supplementary Table 3A)

There was a main effect of block (*p*<0.01), with participants making more optimal responses as the task progressed (4, 3, 2 > 1; *p*<0.05), but no main effects of group or sex (p>0.05). However, there was a block × group interaction (p<0.05), with the controls exhibiting better optimal responding on blocks 2 and 4 than the CUD participants (p<0.05). Thus, the CUD participants exhibited weaker learning rates than the controls, with no differences between males and females.

#### Acquired Equivalence task (Supplementary Table 3B)

On the Acquisition phase, there were no main effects of block, group or sex (p>0.05), and no interactions (p>0.05). On the Transfer phase, there was a main effect of trial type (*p*<0.05), with participants making a greater percentage of errors on the new (Transfer phase) discriminations than the old (Acquisition stage 1) discriminations. There were no main effects of group or sex, or interactions (*p*>0.05). Thus, there was equivalent learning and transfer between groups.

#### Incentive sensitivity, treatment response and dopamine release in CUD

##### CUD Subgroup verification (males only; Supplementary Table 4):

There were significant effects (p<0.05) of: 1) treatment subgroup on vouchers earned (**A**), and 2) DA release subgroup on DA release (**B**). The treatment responders earned more vouchers than the nonresponders and the DA high group exhibited greater DA release than the low group. Thus, subgroup assignments were verified.

##### mIGT Incentive sensitivity (males only):

**Figure 2.**
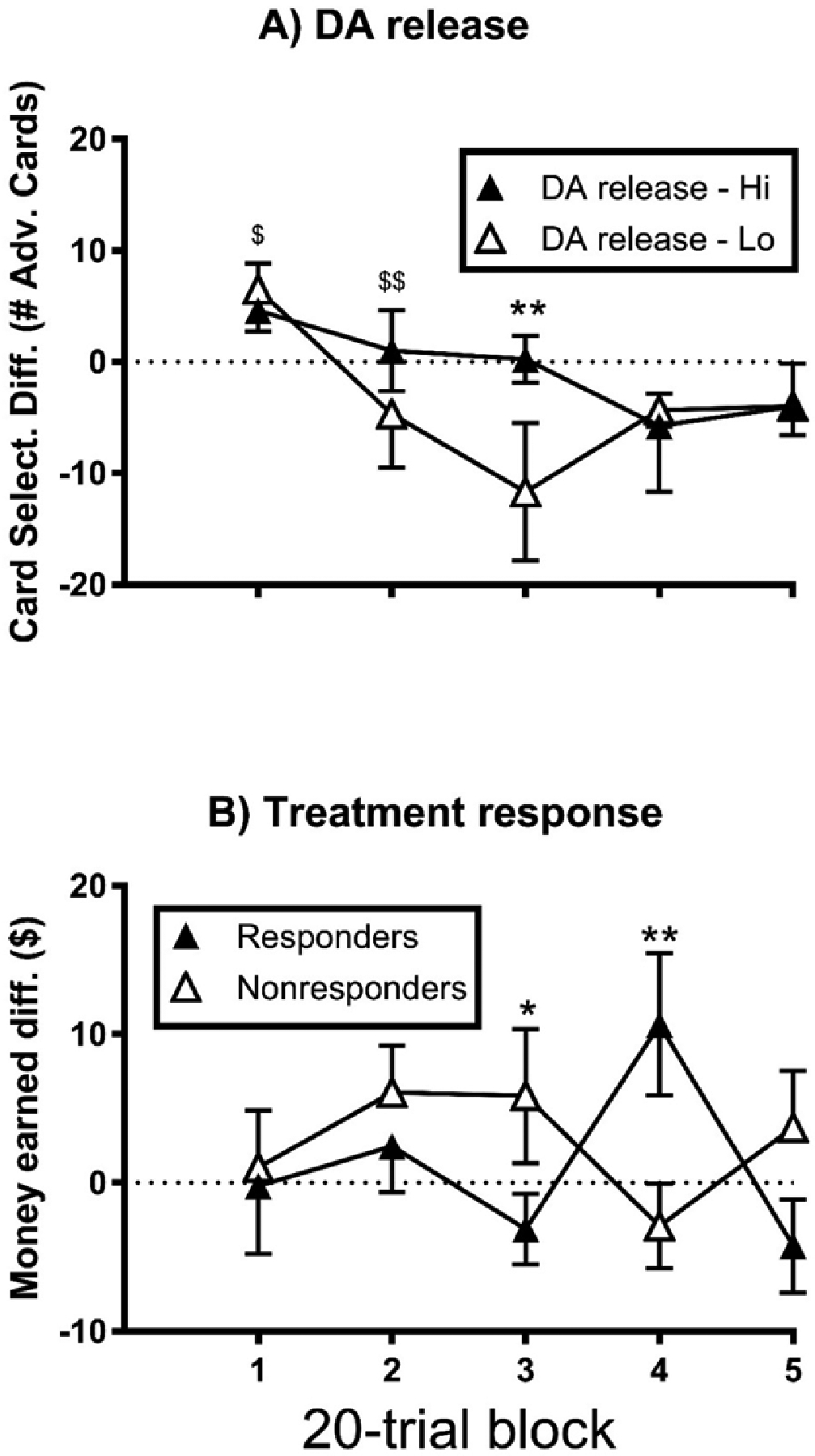
Modified Gambling task incentive sensitivity of male CUD by DA release capacity (**A**) and treatment response (**B**); each error bar represents one SEM; $indicates a significant difference from all other blocks; (p<0.05); $ $indicates a significant difference from block 3 (p<0.05); significant group difference is indicated by: *p<0.05, **p<0.01.

There were no main effects of block or treatment subgroup, nor a block × subgroup interaction (p>0.05) on the GT card selection difference score, and these patterns of significance did not change following covariation for baseline cocaine use (p>0.05; Supplementary Table 5A). For DA release (Supplementary Table 6A), there was a main effect (F_4,44_ = 5.27, η_p_^2^ = 0.32, p<0.01) of block (1>2–5, 2>3; p<0.05). There was no main effect of DA subgroup (p>0.05), but there was a block × subgroup interaction (F_4,44_ = 5.84, η_p_^2^ = 0.35, p<0.01), with the DA high group exhibiting a greater score at block 3 than the DA low group (Figure 2A; p<0.01).

There were no main effects of block (F_4,48_ = 0.35, η_p_^2^ = 0.03, p>0.05) or treatment subgroup (F_1,12_ = 1.50, η_p_^2^ = 0.101 p>0.05), and the block × subgroup interaction was marginal (F_4,48_ = 2.55, η_p_^2^ = 0.18, p=0.05), on the GT money earned difference score. Following covariation for baseline cocaine use, the pattern of significance for main effects remained the same (p>0.05; Supplementary Table 5B). However, the block × subgroup interaction reached significance, with the responders exhibiting a greater difference score at block 4 than the nonresponders (Figure 2b; p<0.01), and vice-versa for block 3 (p<0.05). There was no main effect of block or DA release subgroup, and there was no block × subgroup interaction on the GT money earned difference score (p>0.05; Supplementary Tables 5B and 6B). There was no main effect (p>0.05) of treatment subgroup or DA release subgroup (Supplementary Table 6C) on the GT completion time difference score, and this did not change for treatment subgroup following covariation for baseline cocaine use (Supplementary Table 5C).

Thus, for the male CUD participants, the sensitivity of card selection to monetary incentive was selectively greater (i.e., for one mIGT block) for the high DA releasers than the low releasers. The sensitivity of money earned to monetary incentive was selectively greater for the treatment responders (on one mIGT block), and for the nonresponders (on a different block), once baseline cocaine use was accounted for.

## Discussion

The primary results of this study indicated that: 1) advantageous card selection on the Modified Iowa Gambling Task (mIGT) was increased, but also selectively more sensitive to a real monetary incentive (i.e., decisional incentive sensitivity), in adult participants beginning community reinforcement approach plus vouchers (CRA+V) therapy for cocaine use disorder (CUD), than control participants. 2) Probabilistic category learning (Weather Prediction task) was decreased for CUD participants, relative to controls. 3) Among the male CUD participants, the magnitude of mIGT incentive sensitivity was greater in those who subsequently exhibited relatively greater striatal dopamine release capacity, and (selectively) for those who subsequently responded efficaciously to an incentive-based treatment, relative to those who had relatively lesser dopamine release capacity or were treatment nonresponders.

The finding of increased advantageous card selection on the mIGT overall for these treatment-seeking CUD participants relative to controls was the opposite result of our previous study in nontreatment-seeking CUD participants [15]. It may be driven in part by the disproportionate positive effect of the monetary incentive on the 2^nd^ trial block for the CUD participants (trial block functions were not examined in the previous study). Additionally, the monetary incentive did not improve card selection for any block for the controls. These findings, combined with increased task completion time (i.e., effort incentive sensitivity) overall, are partially consistent with our previous study [15]. The results may suggest that while a monetary incentive may promote greater deliberation and effort on the task in general, it preferentially improves the decision-making of CUD participants (see [50]).

Although the relevance of the specific task block that was affected is unclear, the finding of another (seemingly unrelated) group difference on mIGT card selection at the same block – that female participants were more sensitive to the monetary incentive than the male participants – may suggest that incentives impact card selection only after some initial experience with the task. Regardless, this indicates that sex differences need to be taken into account in studies of decision-making and incentive sensitivity [51].

The finding of decreased probabilistic category learning performance in CUD participants is consistent with previous studies [18,19], but the effects of a monetary contingency on performance of these and other S-R learning tasks by CUD participants (e.g., [20]) have not been studied.

The previously published primary paper [35] from this study showed that in the full sample of CUD participants (males and females), DA release capacity in the ventral striatum (VSt) was greater in subsequent CRA+V responders than non-responders. Additional predictors of outcome from this same sample include clinic attendance early in treatment [52] and implicit beliefs in the positive effects of cocaine (inverse association) [7]. Here, we show in a male subsample that the magnitude of mIGT decision incentive sensitivity was positively associated with VSt DA release capacity, and (to a lesser degree) mIGT outcome incentive sensitivity was associated with CRA+V response; novel findings to our knowledge. This suggests that incentive sensitivity, as derived from the difference between performance under hypothetical and cash incentives on a decision-making task, is a potential cognitive-motivational mechanism by which increased striatal DA function leads to CRA+V outcome success in CUD participants. This adds to the existing literature relating decisional biases to voucher treatment outcome for stimulant use disorder [36,37], and if replicated, may suggest a clinical utility for measuring decisional incentive sensitivity at treatment baseline to aid in intervention choices.

These results converge with findings that neural activation (as measured by fMRI) in multiple brain areas, including the ventral striatum (VSt), in response to partially choice-contingent cash reward anticipation and receipt (Monetary Incentive Delayed Task; Knutson, Westdorp [53)] was elevated in CUD individuals relative to controls [54]. Interestingly, in that study, neural activation in the bilateral thalamus and right caudate, but not VSt, was prospectively associated with percent cocaine-negative urine toxicology in mixed treatment (only 4/22 participants received voucher-based clinical interventions). The caudate is known to be important for S-R learning [55,56], and in an exploratory fashion we found that S-R learning performance, including learning transfer, was associated with post-voucher clinical outcome (Supplementary Figures 2 and 3).

This is interesting since the ability to transfer learning to novel contexts is, arguably, what success in the post-voucher phase of CRA+V depends on. Similarly, the thalamus is important for learning-based attentional function [57], and we previously found that attentional bias towards cocaine-related stimuli was similarly associated with outcome in this same phase of treatment [7]. Thus, distinct brain regions within a network and their corresponding cognitive-motivational functions differentially map on to treatment outcome phases depending on the nature of task and treatment incentives.

It should be noted that despite our primary findings, for some participants the presence of a monetary incentive was associated with decreased advantageous decision-making, similar to real-life high-risk wagering. In the current study (Supplementary Table 7, Supplementary Figure 3), participants whose mIGT performance decreased under the cash condition endorsed greater risk-taking impulsivity in the natural environment on questionnaire measures than those whose performance improved. This provides convergent evidence that task-measured incentive sensitivity is an ecologically-relevant and bi-directional construct that may help understand failure in CRA+V therapy.

There are several limitations to the current study. The small sample size did not permit more advanced statistics that may have addressed all the research questions more parsimoniously. Group differences in sex distribution and depression scores may have influenced the group cross-sectional findings and prevented inclusion of females in the CUD-only analyses. Finally, the $10 credit system for the Cash mIGT condition meant that task losses were not likely to be as salient as winnings.

## Conclusion

Participants seeking treatment for CUD exhibited selectively increased decisional incentive sensitivity, relative to controls, and for male CUD participants, greater incentive sensitivity was associated with greater VSt DA release and better CRA+V outcome. These preliminary data provide a potential model that links DA function, decisional incentive sensitivity and CRA+V outcome that should be explored further in future studies.

## Supplementary Material

ASA-26-011-Supplementary File

## Figures and Tables

**Figure 1.**
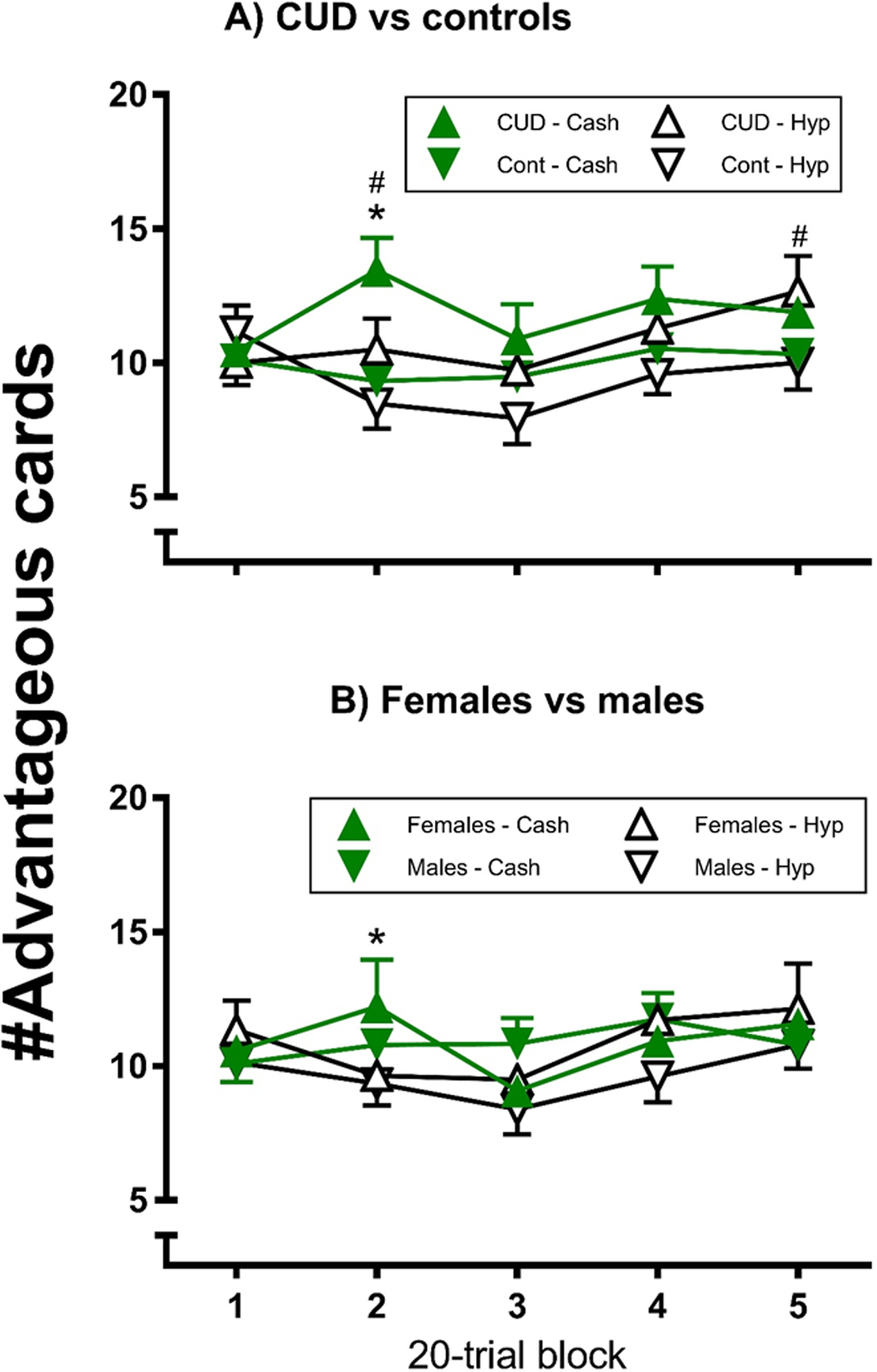
Modified gambling task advantageous card selection by group under Hypothetical and Cash conditions; each error bar represents one SEM; #indicates a significant difference between groups (p<0.05); *indicates a significant difference between conditions for CUD (p<0.05).

**Table 1.** Demographic and clinical characteristics.

	Cocaine users (N=18)		Controls (N=19)		Test value	P value^a^
	M	SD	M	SD		
Age (yrs)	37.2	6.4	33	7.8	t(35) = −1.8	0.08
Education completed (yrs)	13.1	2.4	14.2	2.4	t(35) = 1.6	0.12
Annual household income	26,077	21,484	26,147	19,761	t(35) = 0.1	0.99
**BDI-II total score**	**13.9**	**7.3**	**2.7**	**5**	**t(35) = −5.5**	**<0.001**
RAB total score	4.12	2.89	3.94	1.83	t(33) = −0.2	0.83
BIS total score	68.13	9.56	71.43	8.33	t(28) = 1.0	0.33
	N	%	N	%		
Sex						
**Male**	**15**	**83.3**	**8**	**42.1**	**Fisher’s exact = 0.02**	
**Female**	**3**	**16.7**	**11**	**57.9**		
Race ^d^						
White	8	44.4	7	36.8	χ^2^ (1) = 0.2	0.64
Black	4	22.2	9	47.4	χ^2^ (1) = 2.6	0.11
Hispanic	4	22.2	2	10.5		
Other	2	11.1	1	5.3		
Cocaine						
Age of onset of regular use (yrs)	18.8	3.1				
Frequency (days/past 30)	16.9	8.3				
Amount ($/occasion)	139.8	104.7				
Alcohol	N=12	67%	N=17	90%		
Frequency (days/past 30)	14.8	11	8.8	3.8	t(12.9) = −1.8	0.09
Amount (SDUs/occasion)	3.8	1.8	2.8	1.5	t(27) = −1.8	0.09
Cannabis	N=9	50%	N=12	63%		
Frequency (days/past 30)	6.2	7.5	12.9	8.3	t(19) = 1.9	0.07
**Amount ($/occasion)**	**0.8**	**0.3**	**7**	**2.6**	**t(11.5) = 7.0**	**<0.001**

a bold indicates overall group difference (p<0.05);

d comparisons based on Black vs. not Black and White vs. not White
