# Supplementary material for: Decisional incentive sensitivity is linked to contingency management outcome and striatal dopamine signaling in individuals with cocaine use disorder: a preliminary study
Source: Addict Subst Abus (Middlet). Author manuscript; Available in PMC 2026 Apr 18. (PMC13089948)
Supplement: ASA-26-011-Supplementary File [file NIHMS2164996-supplement-ASA-26-011-Supplementary_File.pdf]

Citation: Vadhan NP, Carpenter KM, Benedict E, Myers CE, Gluck MA, Martinez D, et al. Decisional incentive sensitivity is linked to contingency management outcome and striatal dopamine signaling in individuals with cocaine use disorder: a preliminary study. Addict Subst Abuse. 2026;4(1):1-10.

Supplementary Information

Tasks

Modified Iowa Gambling Task (mIGT)

Participants' individual data were first examined to assess task completion and adequate deck sampling; i.e., at least one choice from each deck. For card selection and money earned, performance was examined as a function of group, payment condition (Cash vs. Hypothetical conditions) and trial block (1-5); i.e., condition × block × group. For completion time, since trial-by-trial data were not recorded, performance was examined as a function of group and payment condition (Cash vs. Hypothetical conditions); i.e., condition × group.

Three indices of incentive sensitivity (decisional, outcome and effort) were created by taking the difference scores between GT performance scores under the 2 monetary conditions (e.g., # advantageous cards$_{Cash}$ - # advantageous cards$_{Hyp}$). To explore the valence of incentive sensitivity, individual participants (cocaine users and controls) were dichotomously classified according to whether or not their 3 mIGT indices increased (positive difference scores) or decreased (negative difference scores) as a function of monetary condition (**Supplementary Table 1**); i.e., whether the monetary incentive promoted more adaptive or more impulsive decision-making.

Supplementary Table 1.

	CUD	Control
**Card selection**		
Positive Valence	n=14 (77.8%)	n=9 (47.4%)
Negative Valence	n=4 (22.2%)	n=10 (52.6%)
**Money earned**		
Positive Valence	n=12 (66.7%)	n=13 (68.4%)
Negative Valence	n=6 (33.3%)	n=6 (31.6%)
**Completion time**		
Positive Valence	n=13 (72.2%)	n=10 (52.6%)
Negative Valence	n=5 (27.8%)	n=9 (47.4%)

The relationship between incentive sensitivity valence (i.e., the directionality of performance change between the hypothetical and cash conditions) and naturalistic risk-taking and impulsivity was examined by comparing the valence subgroups (positive vs negative) on self-report measures of risk-taking (Risk Assessment Battery Total Score) and impulsivity (Barratt Impulsiveness Scale Total Score) via univariate ANOVAs. Group (CUD vs controls) was included as an additional independent variable.

Weather Prediction task

All participants completed the full 200 trials (4 blocks of 50 trials) and were retained for analyses. Mixed (block × group) ANOVAs were used to examine group differences on mean percent of optimal responses.

Acquired Equivalence task

Only data from participants who completed Acquisition stage 3 within 96 trials were analyzed (n=17 cocaine users/17 controls; 20 female/14 male). Mixed-design repeated-measures ANOVAs were used to examine group differences on errors made during: 1) Acquisition stages 1–3 (stage × group) and 2) Transfer phase (trial type × group).

Post-voucher CRA+ V

To explore the relationships between incentive sensitivity and S-R learning and non-incentive-based treatment outcome, CUD participants who continued participation into the post-voucher phase of treatment (n=9) were divided via median split by the number of cocaine metabolite – free urine samples submitted. These subgroups were compared on their incentive sensitivity for the 3 mIGT indices, and performance indices for the WP and AE tasks, via repeated measures ANOVAs as above.

Neuroimaging

ND is the nondisplaceable binding, f_{ND} is the free fraction in the nondisplaceable distribution volume of the brain, B_{max} is the concentration of $D_{2/3}$ receptors (nanomoles per gram of tissue), and K_D is the inverse of the affinity of the radiotracer for the receptor [19]. The region of interest in this study was the limbic striatum since this was the only region found to be related to treatment outcome in the primary paper (Martinez et al., 2009). Activity measurements from the right and left regions were averaged. Identification of the region of interest, motion correction, and PET-to-MRI registration were performed with MEDx (Sensor Systems, Sterling, Va.) as previously described (Martinez et al., 2009).

CRA + V

As described in Martinez et al. (2009), the behavioral treatment consisted of Community Reinforcement Approach plus Vouchers (CRA+V), as previously described by Budney & Higgins (1998). Participants were scheduled to attend the clinic three days a week. On two of the clinic visits, the participants received psychotherapy (CRA), which seeks to develop cocaine avoidance strategies and promote lifestyle changes, such as improving relationships and vocational skills.

The participants' urine was tested for the cocaine metabolite benzoylecgonine on each of the three visits per week. Participants received voucher points for cocaine-negative urine. The points were acquired on an escalating schedule as follows: voucher points ($0.25) started at 10 points for first cocaine-free sample, and each subsequent cocaine-free sample increased the voucher value by 5 points, such that the second consecutive cocaine free urine earned 15 points ($3.75) and the third 20 points ($5.00), etc. Participants also received a bonus of 40 points ($10.00) for every three consecutive cocaine free urine samples (equivalent to a week of abstinence). Abstinence from cocaine was confirmed by on-site urine testing (Abuscreen On-Trak system, Roche Diagnostics) and all urine specimens were sent to the hospital laboratory for confirmation.

Missed clinic appointments and cocaine positive urine samples reset the voucher points to the starting value of 10 points, although participants did not lose the amount of points they earned prior to the time of the value reset. Participants could readjust their voucher points to the maximum amount they had earned prior to the reset by providing 5 consecutive cocaine free urines. The money earned was provided in the form of credit card gift cards, and the use of this money was discussed with the therapist.

Following the 12 weeks of intensive therapy, the cocaine dependent subjects were offered an additional 12 weeks of treatment, in which they were seen in the clinic twice a week, but the voucher system was discontinued. During Phase II, participants were scheduled to attend the clinic twice per week and met once per week with their counselor for approximately 1 hour. The escalating voucher system was discontinued; instead, a New York State lottery ticket was earned for each cocaine-free urine. Counseling sessions were audiotaped and reviewed by the supervising psychologist (KMC) for fidelity and clinical purposes.

Citation: Vadhan NP, Carpenter KM, Benedict E, Myers CE, Gluck MA, Martinez D, et al. Decisional incentive sensitivity is linked to contingency management outcome and striatal dopamine signaling in individuals with cocaine use disorder: a preliminary study. Addict Subst Abuse. 2026;4(1):1-10.

Results

Supplementary Table 2. Modified gambling task performance.

		CUD		Controls		Males		Females		ANOVA results[a, b, c, d]
<u>n</u>		18		19		23		14		
A) Money earned ($)										<u>Main effects and significant interactions</u><br>MC: F(1,32) = 1.01, η_p^2 = 0.03, p=0.32); Blk: F(4,128) = 0.10, η_p^2 = 0.03, p=0.42); Grp: F(1,32) = 1.65, η_p^2 = 0.05, p=0.21); Sex: F(1,32) = 0.21, η_p^2 = 0.01, p=0.65); **Blk × Grp: F(4,128) = 2.89, η_p^2 = 0.08, p=0.03)**
	<u>Block</u>	<u>M</u>	<u>SD</u>	<u>M</u>	<u>SD</u>	<u>M</u>	<u>SD</u>	<u>M</u>	<u>SD</u>	<u>Significant pairwise comparisons</u>[e]
Hypothetical condition	**1**	**-3.8**	**10.6**	**-0.2**	**7.6**	-2.3	10.4	-1.5	7.3	**Cont > Coc (Blk 1 overall)**
	2	0.4	8.2	0.2	7.2	1.3	8	-1.3	6.8	
	3	-2.6	9.2	-4.2	10.2	-2.1	9.4	-5.5	9.9	
	4	-0.7	8	-1.6	7.9	-0.8	7.8	-1.7	8.2	
	5	2	7.7	-1.7	9.7	0.7	9.2	-0.9	8.6	
Cash condition	**1**	**-5**	**6.8**	**0.2**	**7**	-2.1	7.4	-2.7	7.4	
	2	5	6.1	0.1	8	4.1	6.7	-0.1	8.2	
	3	0.1	7.7	-3.5	10	-1	6.7	-3.1	12.1	
	4	-0.6	7.2	0.4	11.6	0.7	9.1	-1.4	10.6	
	5	2.6	8.2	-2.4	11.7	-0.9	11.6	1.6	8.1	
B) Completion time (sec)										<u>Main effects and significant interactions</u><br>**MC: F(1,32) = 21.2, η_p^2 = 0.40, p<0.001)**; Grp: F(1,32) = 0.03, η_p^2 = 0.001, p=0.87); Sex: F(1,32) = 0.04, η_p^2 = 0.001, p=0.85)<br><u>Pairwise comparisons</u>[d]
**Hypothetical condition**	**01-May**	**260.7**	**65.3**	**260.8**	**74**	**256.9**	**62.5**	**267.1**	**69.9**	**Cash > Hypothetical**
**Cash condition**	**01-May**	**328**	**129.7**	**302.6**	**82.7**	**315.4**	**120.6**	**314.3**	**85.5**	

[a] **bold** indicates significant difference (p<0.05); [b] analyses include covariation for condition order; [c] MC=monetary condition, Blk=block, Grp=group, [d] CUD= participants with cocaine use disorder, Cont=controls; [e] only conducted when omnibus ANOVA was significant

Citation: Vadhan NP, Carpenter KM, Benedict E, Myers CE, Gluck MA, Martinez D, et al. Decisional incentive sensitivity is linked to contingency management outcome and striatal dopamine signaling in individuals with cocaine use disorder: a preliminary study. Addict Subst Abuse. 2026;4(1):1-10.

Supplementary Table 3. S-R learning task performance.

	CUD		Controls		Males		Females		ANOVA results[a, b]
n	17		19		22		14		
A) Weather prediction task (% optimal choices)									Main effects and significant interactions<br>**Block: F (2.4,76) = 5.2, η_p^2 = 0.14, p=0.005**; Group: F (1,32) = 3.3, η_p^2 = 0.09, p=0.08; Sex: F (1,32) = 0.4, η_p^2 = 0.01, p=0.55; **Condition × group: F (2.4,76) = 3.9, η_p^2 = 0.11, p=0.02**
Block	M	SD	M	SD	M	SD	M	SD	Significant pairwise comparisons[c]
**1**	**60.6**	**10.5**	**61.0**	**8.7**	**59.9**	**10.3**	**62.3**	**8.0**	**Block 1<2, 3, 4**
**2**	**63.7**	**12.0**	**77.3**	**13.1**	**67.4**	**14.2**	**76.3**	**12.8**	**Cont>CUD**
3	65.7	15.8	72.2	14.4	67.2	14.4	72.2	16.5	
**4**	**65.5**	**10.1**	**77.4**	**12.7**	**70.4**	**11.3**	**73.9**	**15.2**	**Cont>CUD**
B) Acquired Equivalence task									
Acquisition Phase (errors)									Main effects and significant interactions[d]<br>Stage: F (1.8,53.4) = 0.7, η_p^2 = 0.23, p=0.49; Group: F (1,30) = 0.4, η_p^2 = 0.01, p=0.56; Sex: F (1,30) = 2.8, η_p^2 = 0.09, p=0.11
Stage	M	SD	M	SD	M	SD	M	SD	
1	1.9	2.4	1.4	1.3	2.0	2.3	1.1	1.0	
2	1.1	0.9	1.5	2.3	1.6	1.8	0.9	1.6	
3	2.8	5.4	2.4	2.7	3.2	5.0	1.7	2.6	
Transfer Phase (% errors)									Main effects and significant interactions<br>**Trial type: F (1,29) = 4.7, η_p^2 = 0.14, p=0.04**; Group: F (1,29) = 0.4, η_p^2 = 0.01, p=0.54; Sex: F (1,29) = 1.6, η_p^2 = 0.05, p=0.21<br>Significant pairwise comparisons[c]
**"Old"**	**6.9**	**9.8**	**6.5**	**9.2**	**9.3**	**11.4**	**3.2**	**3.6**	**Trial type: New > Old**
**"New"**	**17.2**	**24.2**	**18.6**	**28.7**	**21.1**	**26.7**	**13.6**	**25.9**	

[a] bold indicates significant difference (p<0.05); [b] CUD=participants with cocaine use disorder, Cont = controls; [c] only conducted when omnibus ANOVA was significant; [d] analyses only conducted for Stage 3 solvers

Citation: Vadhan NP, Carpenter KM, Benedict E, Myers CE, Gluck MA, Martinez D, et al. Decisional incentive sensitivity is linked to contingency management outcome and striatal dopamine signaling in individuals with cocaine use disorder: a preliminary study. Addict Subst Abuse. 2026;4(1):1-10.

Supplementary Table S4. CUD subgroup verification (males only).

A) Treatment vouchers earned					
	Treatment responders		Treatment nonresponders		T-test results [a,b]
n	5		10		
	M	SD	M	SD	
**Vouchers ($)**	**635.25**	**165.52**	**47.75**	**38.03**	**TR>TNR; t (4.2) = -7.8, p<0.0001**
B) Ventral striatum dopamine release					
	DA release - High		DA release - Low		T-test results
	8		6		
	M	SD	M	SD	
**BP_{ND}**	**-8.38**	**5.04**	**1.67**	**2.25**	**DA-H<DA-L; t (12) = 4.5, p=0.001**

[a] bold indicates significant subgroup difference (p<0.05); [b] BP_{ND} = binding potential

Citation: Vadhan NP, Carpenter KM, Benedict E, Myers CE, Gluck MA, Martinez D, et al. Decisional incentive sensitivity is linked to contingency management outcome and striatal dopamine signaling in individuals with cocaine use disorder: a preliminary study. Addict Subst Abuse. 2026;4(1):1-10.

Supplementary Table S5. CRA+V response and incentive sensitivity (CUD males only).

	Responders ®		Nonresponders (NR)		ANOVA results[a, b, c, d]
n	5		10		
A) MGT[e] advantageous card selection difference score (#)	M	SD	M	SD	Main effects and interactions<br>Blk: $F(4,44) = 0.55$, $\eta_p^2 = 0.48$, p=0.70); Grp: $F(1,11) = 0.16$, $\eta_p^2 = 0.01$, p=0.70); Blk × Grp: $F(4,44) = 0.28$, $\eta_p^2 = 0.03$, p=0.89)
Block					Significant pairwise comparisons [c]
1	7.60	2.41	5.60	7.34	
2	.40	7.93	-0.90	12.81	
3	-5.60	6.99	-5.25	14.85	
4	-4.80	16.53	-4.80	10.25	
5	-6.40	8.53	-1.80	7.86	
B) mIGT money earned difference score ($)					Main effects and interactions<br>Blk: $F(4,44) = 0.76$, $\eta_p^2 = 0.06$, p=0.56); Grp: $F(1,11) = 3.74$, $\eta_p^2 = 0.25$, p=0.08); **Blk × Grp: $F(4,44) = 3.35$, $\eta_p^2 = 0.23$, p=0.02**)
Block					Significant pairwise comparisons [c]
1	-0.22	10.23	1.04	12.13	
2	2.47	6.97	6.08	9.99	
**3**	**-3.11**	**5.31**	**5.85**	**14.25**	**NR>R**
**4**	**10.67**	**10.70**	**-2.91**	**9.01**	**R>NR**
5	-4.25	7.03	3.74	12.02	
C) mIGT completion time difference score (sec)	91.40	110.77	54.30	150.72	Main effect<br>Grp: $F(1,11) = 0.06$, $\eta_p^2 = 0.005$, p=0.81)

[a] bold indicates significant difference (p<0.05); [b] analyses include covariation for condition order; [c] Blk=block, Grp=group, [d] only conducted when omnibus ANOVA was significant; [e] mIGT = modified gambling task

Citation: Vadhan NP, Carpenter KM, Benedict E, Myers CE, Gluck MA, Martinez D, et al. Decisional incentive sensitivity is linked to contingency management outcome and striatal dopamine signaling in individuals with cocaine use disorder: a preliminary study. Addict Subst Abuse. 2026;4(1):1-10.

Supplementary Table S6. Dopamine release and incentive sensitivity (CUD males only).

		DA release - High		DA release - Low		ANOVA results[a, b, c, d]
n		8		6		
A) mIGT[e] advantageous card selection difference score (#)						Main effects and interactions<br>**Blk: F(4,44) = 5.27, η_p^2 = 0.32, p=0.001)**; Grp: F(1,11) = 0.07, η_p^2 = 0.01, p=0.79); **Blk × Grp: F(4,44) = 5.84, η_p^2 = 0.35, p=0.001)**
Block		M	SD	M	SD	Significant pairwise comparisons [c]
	1	4.63	5.29	6.50	5.75	
	2	1.00	10.25	-4.67	11.78	
	**3**	**.25**	**5.90**	**-11.67**	**15.15**	**$DA_{high} > DA_{low}$**
	4	-5.75	16.65	-4.33	3.67	
	5	-4.00	7.33	-4.00	9.47	
B) mIGT money earned difference score ($)						Main effects and interactions<br>Blk: F(4,44) = 0.41, η_p^2 = 0.36, p=0.80); Grp: F(1,11) = 3.41, η_p^2 = 0.24, p=0.09); Blk × Grp: F(4,44) = 0.58, η_p^2 = 0.05, p=0.68)
Block		M	SD	M	SD	Significant pairwise comparisons [d]
	1	0.49	9.56	1.33	14.80	
	2	3.04	8.22	6.03	10.65	
	3	0.43	9.46	4.60	16.93	
	4	0.73	12.20	5.41	9.24	
	5	2.82	12.55	-2.76	9.10	
C) mIGT completion time difference score (sec)		127.00	140.48	2.50	71.12	Main effects<br>Grp: F(1,14) = 1.24, η_p^2 = 0.08, p=0.28)

[a] bold indicates significant difference (p<0.05); [b] Blk=block, [c] Grp=group, [d] only conducted when omnibus ANOVA was significant; [e] mIGT = modified gambling task

Citation: Vadhan NP, Carpenter KM, Benedict E, Myers CE, Gluck MA, Martinez D, et al. Decisional incentive sensitivity is linked to contingency management outcome and striatal dopamine signaling in individuals with cocaine use disorder: a preliminary study. Addict Subst Abuse. 2026;4(1):1-10.

Supplementary Table 7. Incentive sensitivity valence and self-reported risk-taking/impulsivity.

	Valence – Positive (VP)				Valence – Negative (VN)				ANOVA results[a, b, c]
	CUD		Cont		CUD		Cont		Main effects and interactions
	M	SD	M	SD	M	SD	M	SD	
A) mIGT[d] decisional incentive sensitivity (card selection)									
**Risk Assessment Battery scores**	**3.23**	**1.74**	**3.50**	**1.77**	**7.00**	**4.24**	**4.30**	**1.89**	**ISV: F(1,30) = 7.12, ηp2 = 0.19, p=0.02)**, **VN>VP**; Grp: F(1,30) = 2.01, ηp2 = 0.06, p=0.17); ISV × Grp: F(1,30) = 2.95, ηp2 = 0.09, p=0.096)
Barratt Impulsiveness Scale	66.67	8.48	73.14	9.19	72.50	12.61	69.71	7.68	ISV: F(1,25) = 0.002, η_p^2 = 0.00, p=0.97); Grp: F(1,25) = 0.38, η_p^2 = 0.02, p=0.54); ISV × Grp: F(1,25) = 1.53, η_p^2 = 0.23, p=0.23)
B) mIGT outcome incentive sensitivity ($ earned)									
Risk Assessment Battery scores	3.46	1.51	4.00	1.60	5.33	4.41	3.83	2.40	ISV: F(1,30) = 0.67, ηp2 = 0.02, p=0.42); Grp: F(1,30) = 0.25, ηp2 = 0.008, p=0.62); ISV × Grp: F(1,30) = 1.58, ηp2 = 0.05, p=0.22)
**Barratt Impulsiveness Scale**	**64.50**	**8.62**	**67.25**	**4.90**	**74.17**	**8.38**	**77.00**	**9.03**	**ISV: F(1,25) = 9.98, ηp2 = 0.29, p=0.004), VN>VP**; Grp: F(1,25) = 0.88, ηp2 = 0.03, p=0.36); ISV × Grp: F(1,25) = 0.00, ηp2 = 0.00, p=0.99)
C) mIGT effort incentive sensitivity (completion time)									
Risk Assessment Battery scores	4.75	3.14	4.20	2.10	2.60	1.52	3.63	1.51	ISV: F(1,30) = 0.97, ηp2 = 0.03, p=0.33); Grp: F(1,30) = 0.08, ηp2 = 0.003, p=0.78); ISV × Grp: F(1,30) = 0.88, ηp2 = 0.03, p=0.36)
Barratt Impulsiveness Scale	69.82	8.66	71.88	6.58	64.40	11.39	70.83	10.91	ISV: F(1,25) = 0.27, ηp2 = 0.01, p=0.61); Grp: F(1,25) = 1.45, ηp2 = 0.06, p=0.24); ISV × Grp: F(1,25) = 0.42, ηp2 = 0.02, p=0.52)

[a] bold indicates significant difference (p<0.05); [b] ISV=Incentive sensitivity valence subgroup, [c] Grp=group, [d] mIGT = modified gambling task

There was a main effect of decisional (card selection) incentive sensitivity valence subgroup on RAB total scores (Figure S1), with the negative subgroup exhibiting higher scores than the positive subgroup (p<0.05), but not for BIS scores (p>0.05). There was a main effect of outcome (money earned) incentive sensitivity valence subgroups on BIS total scores (Figure S3B), with the negative subgroup exhibiting higher scores than the positive subgroup (p<0.05), but not for RAB scores (p>0.05). There was no main effect of the outcome incentive sensitivity subgroups on RAB total scores, and no main effect of decisional incentive sensitivity subgroups on BIS total scores (p>0.05). There were no main effects of effort (completion time) incentive sensitivity subgroups on these scores, and no subgroup × group interactions for any of the three incentive sensitivity valence variables (p>0.05). Thus, participants whose mIGT card selection became more adaptive under the monetary contingency endorsed less frequent naturalistic risk-taking than those whose card selection became riskier, and those whose mIGT money earned increased under the monetary contingency endorsed lower levels of naturalistic impulsivity than those whose money earned decreased.

Citation: Vadhan NP, Carpenter KM, Benedict E, Myers CE, Gluck MA, Martinez D, et al. Decisional incentive sensitivity is linked to contingency management outcome and striatal dopamine signaling in individuals with cocaine use disorder: a preliminary study. Addict Subst Abuse. 2026;4(1):1-10.

Supplementary Figure 1. Weather prediction task performance as a function of post-voucher treatment response (male CUD participants only).

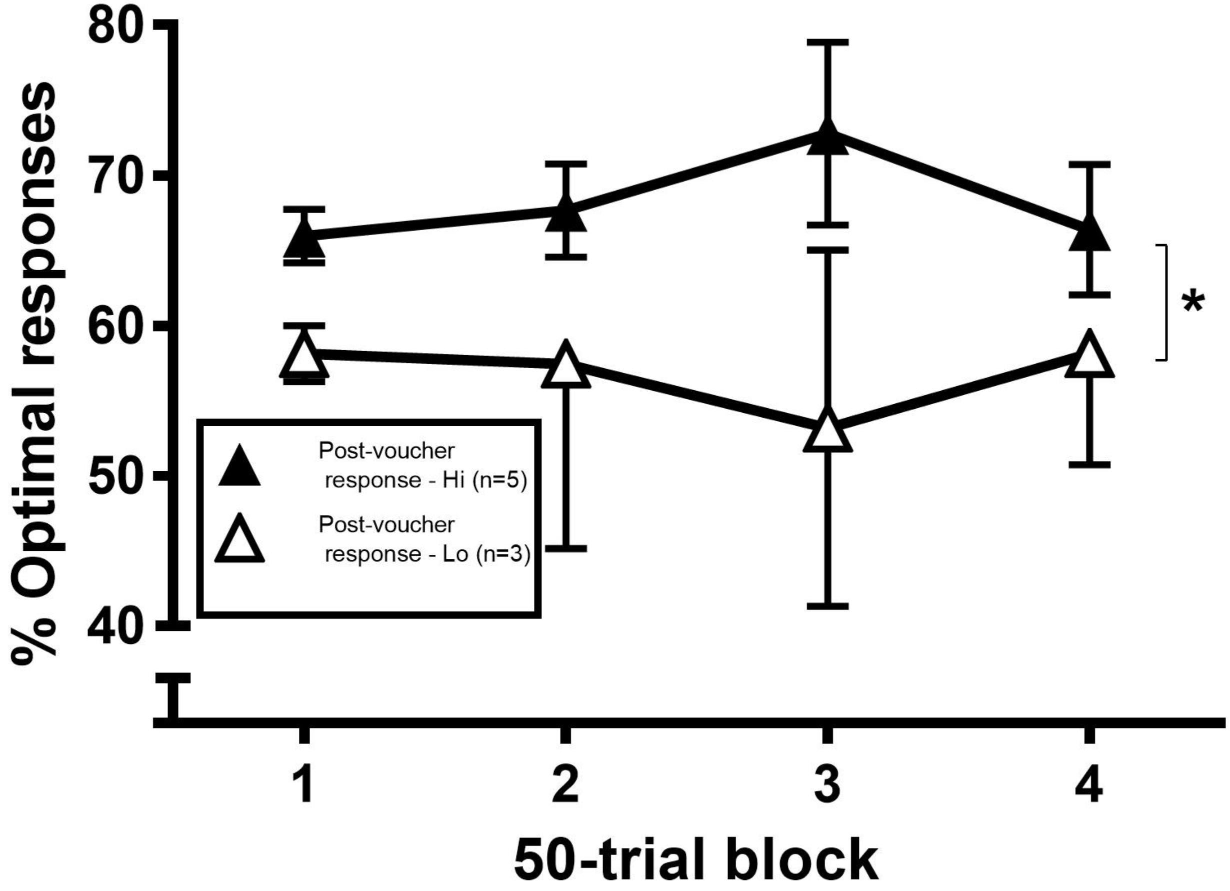

Citation: Vadhan NP, Carpenter KM, Benedict E, Myers CE, Gluck MA, Martinez D, et al. Decisional incentive sensitivity is linked to contingency management outcome and striatal dopamine signaling in individuals with cocaine use disorder: a preliminary study. Addict Subst Abuse. 2026;4(1):1-10.

Supplementary Figure 2. Acquired equivalence task performance as a function of post-voucher treatment response (male CUD participants only).

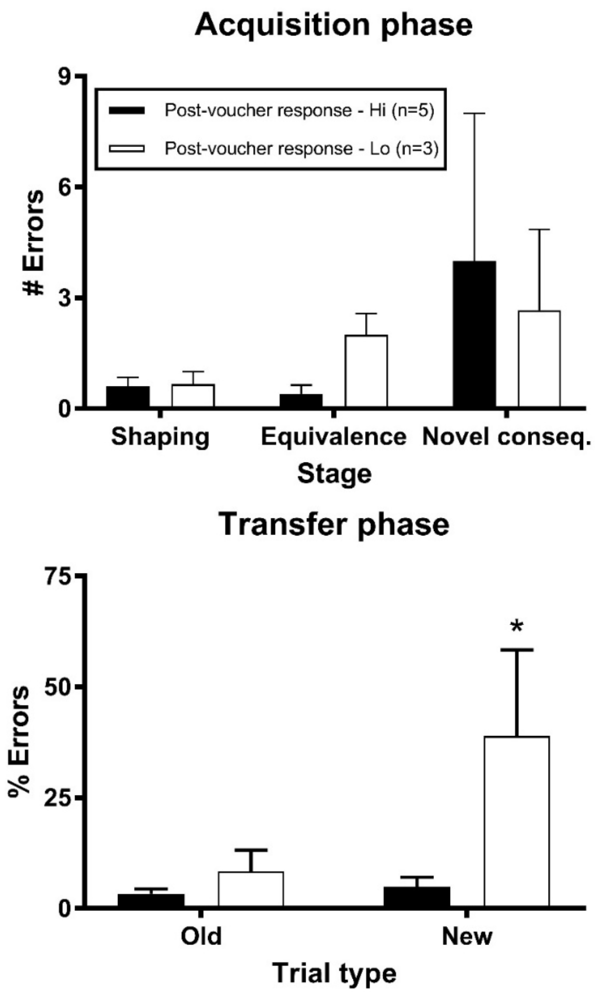

Citation: Vadhan NP, Carpenter KM, Benedict E, Myers CE, Gluck MA, Martinez D, et al. Decisional incentive sensitivity is linked to contingency management outcome and striatal dopamine signaling in individuals with cocaine use disorder: a preliminary study. Addict Subst Abuse. 2026;4(1):1-10.

Supplementary Figure 3. Risk assessment battery scores by incentive sensitivity valence and group.

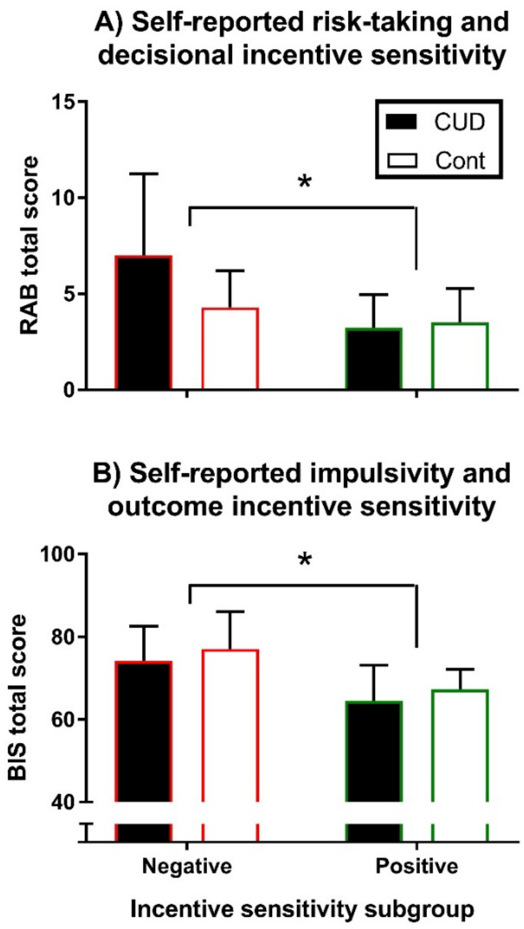

Each error bar represents one SEM; *Significant group difference between negative and positive mIGT incentive sensitivity valence subgroups (p<0.05).
